# Does MMPI assessed at medical school admission predict psychological problems in later years?

**DOI:** 10.1186/s13104-019-4524-5

**Published:** 2019-08-05

**Authors:** Kulvadee Thongpibul, Pairada Varnado, Nahathai Wongpakaran, Tinakon Wongpakaran, Pimolpun Kuntawong, Danny Wedding

**Affiliations:** 1 Department of Psychology, Faculty of Humanitites, Chiang Mai University, Chiang Mai, Thailand; 2 Department of Psychiatry, Faculty of Medicine, Chiang Mai University, Chiang Mai, Thailand; 3 School of Humanistic and Clinical Psychology, Saybrook University, Oakland, CA USA

**Keywords:** MMPI, Medical students, Psychological distress, Perceived stress, Depression

## Abstract

**Objective:**

Psychological distress among medical students is related to personality. The Minnesota Multiphasic Personality Inventory (MMPI) is a common instrument used to assess personality and psychological problems during the medical school admission process in Thailand. The purpose of this study was to examine how the MMPI can predict medical students’ psychological problems including perceived stress, anxiety, depression, interpersonal difficulties as well as self-esteem in later years.

**Results:**

Anxiety and depressive symptoms were predicted by the psychopathic deviation, psychasthenia, and schizophrenia scales of the MMPI, while perceived stress was predicted by schizophrenia scale of MMPI. Social introversion predicted interpersonal difficulties. No MMPI scale was found to predict self-esteem.

**Electronic supplementary material:**

The online version of this article (10.1186/s13104-019-4524-5) contains supplementary material, which is available to authorized users.

## Introduction

Medical education is challenging and demanding. Apart from intellectual ability, personality traits are believed to be important factors that affect medical school performance, psychological adaptation, motivation in medical education and psychological problems, which may interfere with study [1–3].

Assessing psychological problems and personality traits is a routine practice as a part of the medical students’ admission process. The Minnesota Multiphasic Personality Inventory (MMPI) is an instrument that has been used globally to assess personality and psychological issues. Poor academic performance was related to the MMPI scales of hysteria, psychopathic deviate, and schizophrenia [4].

Currently, medical schools in Thailand use the MMPI as a tool to assess psychopathology during the student selection process. Although MMPI results typically are not used for admission decision, they help direct the attention of the interview committee to applicants who score high on certain MMPI scales.

Common psychological problems among medical students include anxiety, depression, and suicidal ideation, all of which relate to personality [2–5]. Evidence has shown that Thai medical students tended to have higher scores on most subscales of the MMPI by the third year of medical school [6]. This suggests that psychopathology can also relate to personality traits.

On the other hand, positive attributes such as self-esteem play important roles not only in medical performance [7], but also in influencing a student’s sense of well-being [8]. Self-esteem has been shown to be related to extraversion and neuroticism [9], and some items of MMPI were drawn to form a self-esteem content scale in the MMPI-2 [10]. However, to the best of our knowledge, the relationship between MMPI clinical scales and self-esteem has not yet been explored.

This study examined Thai medical student’s MMPI personality profiles, assessed during the admission process at a Chiang Mai medical school, in predicting changes regarding psychological problems and self-esteem during subsequent years. We hypothesized that some MMPI clinical scales would predict these changes. If so, employing the MMPI during the admission process would help to identify those students at risk of incurring psychological problems or diminished levels of self-esteem.

## Main text

### Methods

#### Design

This study employed an observational and prospective design to explore using the MMPI to predict both positive and negative psychological outcomes.

#### Participants

Participants were medical students who were administered the MMPI as part of the medical school admission process. Two hundred and fifty five students, who passed the written entrance examination for the medical school, completed the MMPI before the admission interview. The MMPI results were then provided to the interview committee as additional data to aid in the committee’s decision-making. Of the 250 students who was ultimately admitted into the medical school, 203 students participated in this study; however 201 students completed various psychological measures that assessed self-esteem, perceived stress, perceived social support, interpersonal problems, and psychological symptoms (Time 1). Participants were followed-up for changes in previously noted psychological variables and asked to complete the same measures when they were in their third year of medical school (Time 2). One hundred and ninety-six students completed the psychological measures during their third year of medical study (Additional file 1: Figure S1). Mean age of the participants was 18.64 years old (SD = .59), and 60.7% were female.

### Measurement

#### MMPI

The MMPI is a standardized psychometric test of adult personality and psychopathology. The version used for the study was the MMPI developed by Hathaway & McKinley [11] and translated into Thai language by Kasemsak Poomsrikeo [12]. The MMPI has 10 clinical scales, i.e., (1) hypochondriasis—Hs, (2) depression—D, (3) hysteria—Hy, (4) psychopathic deviation—Pd, (5) femininity/masculinity—F/M, (6) paranoia—Pa, (7) psychasthenia—Pt, (8) schizophrenia—Sc, (9) mania—Ma, and (10) social introversion—Si. In addition, the MMPI has scales designed to detect when test-takers are under-reporting or downplaying psychological symptoms, i.e., lying (L), defensiveness (K) and faking (F). The MMPI Thai version has been tested in and shown to have discriminant validity in that the subscale scores were significantly higher in heroin than in nonheroin addicted juveniles group (*p *< .01) [12].

The Thai adaptation team concluded that the American norms were acceptable to use with Thai clients because prior work indicated that normal samples in Thailand scored in a similar range as the American subjects on the MMPI scales [6].

#### Perceived stress scale (PSS)

This scale measures how an individual perceives stress. It is a 10-item self-report instrument that uses a 5-scale Likert format 0 (never) to 4 (very often), with total score ranges from 0 to 40 [13]. Higher scores indicate greater perceived stress. The Thai version of the PSS-10 demonstrated good reliability and validity [14] and the present study yielded a Cronbach’s alpha of .85.

#### Outcome inventory (OI-21)

The Outcome Inventory is a self-rating questionnaire that measures four common mental health problems: anxiety, depression, interpersonal difficulties and somatic complaints. It includes 21 questions assessed using Likert scales that range from 1 (not at all) to 5 (very much). A higher score indicates a higher level of psychopathology [15]. In the present study, only anxiety, depression and interpersonal difficulties were used and yielded a Cronbach’s alpha of .89.

#### Rosenberg self-esteem scale (RSES)

The RSES is a self-rating questionnaire that measures self-worth or self-esteem. It includes 10 questions assessed using Likert scales that range from 1 (strongly disagree) to 4 (strongly agree). The Thai RSES demonstrated good validity and reliability, and the present study yielded a Cronbach’s alpha of .88 [16].

#### Statistical analysis

Sociodemographic data such as sex and age were described by percentage and mean (SD). Pearson’s or Spearman’s rank correlation coefficients were used to examine the linear relationship between MMPI scales and outcome measures, i.e., anxiety, depression, perceived stress and self-esteem. Two-sample t-tests, ANOVA, Kruskal–Wallis or Mann–Whitney U tests were used to compare outcome measures between time 1 and time 2, as appropriate.

Because time serves as an important predictor in this case, multilevel linear models were applied to assess the relationship between the independent variables of interest including time and MMPI scales, while dependent (outcome) variables included anxiety, depression, interpersonality difficulties, perceived stress, and self-esteem. Fixed effects models were fitted first and then random intercepts and slopes were introduced using maximum likelihood methods. Intercepts and slopes for each subject were allowed to vary for the MMPI scales. All data were analyzed using IBM SPSS 22.0. The data were normally distributed (skewness and kurtosis between − 2 and + 2), and the final best fitting models are presented below.

### Results

Table 1 shows the different outcomes between time 1 (on admission) and time 2 (year 3 of the study). No differences were found among psychological symptoms. However, self-esteem and perceived stress scale scores were significantly higher in year 3 than at admission (p < .05 and p < .001, respectively).

**Table 1:** Comparing outcome between time 1 and time 2

	Time 1			Time 2			t	n	*p* value
	n	Mean (SD)	Min–max	n	Mean (SD)	Min–max			
Anxiety	196	13.56 (3.6)	6–24	236	13.08 (4.0)	6–23	1.55	191	.122
Depression	196	7.16 (2.0)	5–15	236	7.16 (2.5)	5–24	.79	191	.433
Interpersonal difficulties	196	7.48 (2.1)	4–16	235	7.84 (2.5)	4–19	− 1.37	190	.173
Self-esteem	196	32.71 (3.7)	17–40	235	34.17 (4.0)	19–40	− 2.09	190	.038
Perceived stress	196	13.55 (4.4)	0–26	229	14.31 (5.3)	0–32	− 4.89	185	.000

*SD* standard deviation, *n* number, *min–max* minimum–maximum, *t* t-statistics

Additional file 2: Table S1 shows the mean and standard deviation of the MMPI scales, which were mostly in an acceptable range (40 to 60), except for defensiveness, which was slightly, but predictable, higher.

Table 2 shows the correlation coefficients between MMPI scales and outcome measures at time 1 and time 2. The correlation coefficients ranged from − .141 to − .285. The depression scale did not significantly correlate with depressive symptoms either at time 1 or at time 2.

**Table 2:** Bivariate Correlation between MMPI scales and Outcome measures at time 1 and time 2

Scale	Time 1					Time 2				
	Anxiety	Dep	Int	SES	pss	Anxiety	Dep	Int	pss	SES
Lie	− .126	− .061	.035	.108	− .069	− .141*	− .038	− .053	− .084	.132
Fake	.059	.043	.094	− .141	.006	.023	.060	.205**	.039	− .142*
Defensive	− .102	− .101	− .050	.224**	− .128	− .285**	− .172*	− .082	− .243**	.171*
Hs	.038	.021	.041	.006	− .006	− .045	− .025	− .003	− .057	− .052
D	− .062	− .094	.122	− .111	− .006	.025	.011	.177*	.012	− .139
Hy	− .030	.008	− .010	.043	− .075	− .154*	− .157*	− .072	− .211**	.116
Pd	− .127	− .051	− .002	.073	− .088	− .226**	− .142*	.056	− .199**	.075
M/F	.073	− .046	.001	− .093	− .018	.054	.072	.087	− .022	− .020
Pa	.058	.148	.051	− .084	.145	.161*	.125	.136	.117	− .059
Pt	.105	.040	.045	− .145	.117	− .078	− .080	.114	− .054	− .009
Sc	.158*	.179*	.083	− .071	.141	.048	.061	.132	.073	− .118
Hy	.044	.110	− .043	− .078	.070	− .020	.009	.049	− .006	.111
Si	.103	.130	.247**	− .175*	.138	.098	.025	.196**	.089	− .215**

*Dep* depression, *Int* interpersonal difficulties, *pss* overall perceived stress, *SES* self-esteem

* p < .05, ** p < .01

In terms of sex, no significant differences were found on outcome measures except for anxiety. On the MMPI scales, significant differences were found for hypochondriasis, depression, hysteria, masculinity/femininity and psychasthenia.

Table 3 shows that after sex and age were accounted for, four MMPI scales related to the change in outcome scores: psychopathic deviation, psychasthenia, schizophrenia, and social introversion. Changes in the self-esteem scales were not predicted by any MMPI scales.

**Table 3:** Predictors of MMPI scales on each outcome measure

Variable	Anxiety			Depression			Interpersonal difficulties			Self-esteem			Perceived stress		
	F	df	p-value	F	df	p-value	F	df	p-value	F	df	p-value	F	df	p-value
Time	3.281	161.95	.072	1.126	161.67	.290	44.395	161.45	.214	21.436	160.34	.000	2.303	161.81	.131
Lie	.001	146.62	.979	.863	146.38	.354	.041	146.48	.841	.181	148.37	.671	2.535	146.10	.113
Fake	2.997	146.99	.085	1.202	146.74	.275	.729	146.79	.394	.019	146.95	.892	3.795	146.43	.053
Defensive	2.932	146.66	.089	.782	146.42	.378	.555	146.52	.458	1.458	147.70	.229	3.678	146.14	.057
Hs	1.144	146.71	.286	1.278	146.47	.260	.069	146.56	.793	.391	146.58	.532	1.126	146.19	.290
D	.006	146.99	.940	.834	146.74	.363	.359	146.79	.550	1.473	149.42	.226	.002	146.43	.961
Hy	.104	146.67	.747	.680	146.42	.411	.033	146.52	.856	.221	146.80	.638	3.487	146.14	.063
Pd	*5.984*	147.48	*.015*	1.709	147.23	.193	.126	147.20	.723	.352	149.19	.553	3.076	146.87	.081
M/F	1.330	146.70	.250	.088	146.46	.767	.142	146.55	.707	.374	145.93	.542	.232	146.18	.630
Pa	3.274	146.76	.072	3.182	146.51	.076	1.965	146.59	.163	.331	146.06	.566	3.496	146.22	.063
Pt	2.108	146.66	.148	*5.044*	146.42	*.026*	1.787	146.52	.183	.226	147.78	.635	.130	146.14	.719
Sc	*10.116*	146.65	*.002*	*10.208*	146.41	*.002*	.256	146.51	.614	.918	145.77	.339	*7.912*	146.13	*.005*
Hy	1.867	146.62	.174	.029	146.38	.865	.010	146.48	.922	.987	147.51	.322	1.339	146.10	.249
Si	.002	146.78	.969	.816	146.53	.368	*9.994*	146.61	*.002*	.522	147.30	.471	.018	146.24	.894

*F* F-statistics, *df* degree of freedom

Significant values are in italics

### Discussion

The aim of the present study was to examine the predictive validity of the MMPI at admission against subsequent psychological problems and self-esteem. Even though no significant differences were found concerning anxiety and depression between time 1 (year 1) and time 2 (year 3), we found the schizophrenia, psychopathic deviation, and psychasthenia scales predicted individual changes among these psychological distress scores. Overall, the schizophrenia scale was the best predictor of negative mental health. As expected, changes in interpersonal difficulties were predicted by social introversion MMPI scale. Interestingly, depression was not predicted by the MMPI depression scale. We suspect that the participants were aware of the role of the MMPI in the medical school admission process and hence tended to present themselves in a favorable light and underreport their symptoms for fear that they might not be granted entry into the medical school or be labeled as mentally unhealthy. This assumption is supported by the high correlation between the defensive scale and psychological problems. Notably, even though no difference was found regarding the means for anxiety and depression for the whole group, schizophrenia, psychopathic deviation and psychasthenia were shown to predict changes for participants. In addition, the schizophrenia, psychopathic deviation and psychasthenia scales have been shown to predict behavioral problems such as drug abuse.

Related research showed that perceived stress was significantly higher in the medical students in the third year comparing to the first year [17]. However, to the best of our knowledge, no study has focused on associations between MMPI scales and changes in perceived stress. We found the schizophrenia scale predicted changes in perceived stress. Low scores on reality testing of the schizophrenia scale has been shown to relate to low scores on measures of emotional intelligence [18] and medical students with higher emotional intelligence experienced lower levels of stress [17].

High scores on the MMPI schizophrenia scale relate to unusual beliefs and eccentric behaviors, and do not necessarily mean that participants met the criteria of schizophrenia; instead, high scores (e.g., t-score of 65 or above) suggest personality processes associated with increased liability to developing schizophrenia-related illness [19]. High scores on the schizophrenia scale most often result from poor social skills, limited skill in judgment, and impaired logical thinking [20], making those with high scores more likely to experience psychological problems.

In medical settings, where the MMPI is most appropriately used, patients with high scores on schizophrenia, psychopathic deviation and psychasthenia scales should be monitored to allow for early intervention to prevent further psychological problems. Medical students in higher clinical years (years 4–6) tend to have more psychological problems due to clinical training encounter [21]. Hence, further investigation that includes higher clinical years may provide a more comprehensive picture of the value of the MMPI in predicting psychological problems encountered by medical students.

### Conclusion

The MMPI predicted higher scores on measures of anxiety, depression, interpersonal difficulties and perceived stress. Schizophrenia, psychopathic deviation and psychasthenia were the MMPI scales found to be most predictive, as found in other studies. It may be useful to use the MMPI to identify students at risk of developing psychopathology so that appropriate interventions could be provided as early as possible.

## Limitations

The MMPI, administered during the medical school admissions process, was likely to be biased because of the demand characteristics of the situation. Even though biases are somewhat corrected using regression analysis, it is rather difficult to assess the influence of situational variables. In addition, the results may not be generalized to other settings, because MMPI scores obtained on admission may not reflect the true personality and psychopathology of the respondents. Finally, an older version of the MMPI was used for this study, and further exploration using a more recent version of the test is warranted.

## Additional files

**Additional file 1: Figure S1.** Flow chart of the study.

**Additional file 2: Table S1.** MMPI scale scores on admission.

## Data Availability

The datasets used and/or analyzed during the current study are available from the corresponding author upon reasonable request as data sharing is subject to Ethics Office approval.

## Abbreviations

MMPI: Minnesota Multiphasic Personality Inventory
Hs: hypochondriasis
D: depression
Hy: hysteria
Pd: psychopathic deviate
M/F: masculinity/femininity
Pa: paranoia
Pt: psychasthenia
Sc: schizophrenia
Hy: hypomania
Si: social introversion
