# Supplementary figures and images for: Does MMPI assessed at medical school admission predict psychological problems in later years?
Source: BMC Res Notes. 2019 Aug 5;12:480. doi: 10.1186/s13104-019-4524-5 (PMC6683350; doi:10.1186/s13104-019-4524-5)

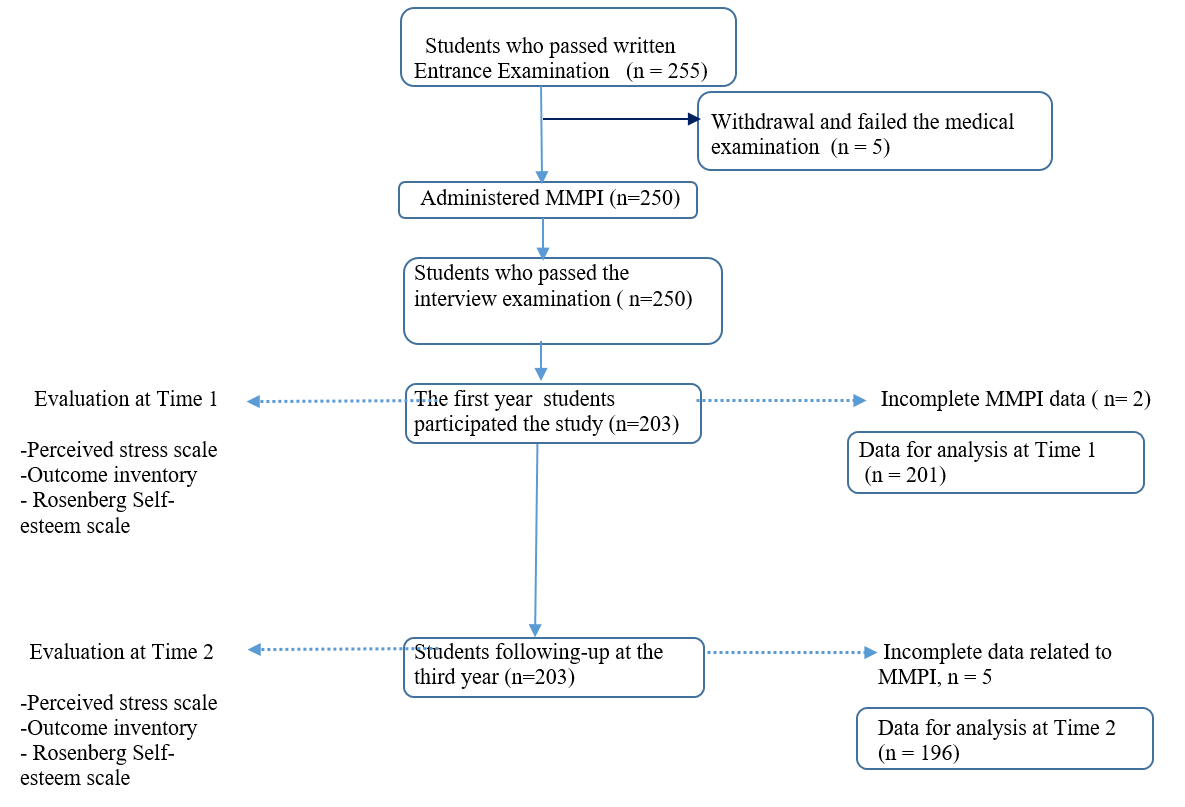

Supplement: Supplementary file 1 — Additional file 1: Figure S1. Flow chart of the study. [file 13104_2019_4524_MOESM1_ESM.tif]
