# Supplementary material for: Does MMPI assessed at medical school admission predict psychological problems in later years?
Source: BMC Res Notes. 2019 Aug 5;12:480. doi: 10.1186/s13104-019-4524-5 (PMC6683350; doi:10.1186/s13104-019-4524-5)
Supplement: Supplementary file 2 — Additional file 2: Table S1. MMPI scale scores on admission. [file 13104_2019_4524_MOESM2_ESM.docx]

| Table S1 MMPI scale scores on admission | | | | | |
| --- | --- | --- | --- | --- | --- |
| Scale | n | min | max | Mean | SD |
| Lie | 201 | 47 | 77 | 59.90 | 6.13 |
| Fake | 201 | 43 | 83 | 55.39 | 7.01 |
| Defensive | 201 | 36 | 78 | 61.15 | 8.46 |
| Hs | 201 | 38 | 78 | 54.82 | 7.20 |
| D | 201 | 39 | 102 | 56.85 | 8.53 |
| Hy | 201 | 37 | 71 | 55.83 | 6.45 |
| Pd | 201 | 31 | 81 | 54.59 | 8.26 |
| M/F | 201 | 38 | 78 | 58.77 | 7.73 |
| Pa | 201 | 39 | 76 | 56.68 | 7.29 |
| Pt | 201 | 32 | 84 | 57.59 | 7.77 |
| Sc | 201 | 38 | 85 | 60.13 | 6.85 |
| Hy | 201 | 38 | 83 | 60.74 | 8.15 |
| Si | 201 | 32 | 69 | 49.32 | 6.34 |
| n = number, min = minimum, max = maximum, SD = standard deviation | | | | | |
